# The Relationship of Sugar to Population-Level Diabetes Prevalence: An Econometric Analysis of Repeated Cross-Sectional Data

**DOI:** 10.1371/journal.pone.0057873

**Published:** 2013-02-27

**Authors:** Sanjay Basu, Paula Yoffe, Nancy Hills, Robert H. Lustig

**Affiliations:** 1 Stanford Prevention Research Center, Department of Medicine, Stanford University, Palo Alto, California, United States of America; 2 Department of Integrative Biology, University of California, Berkeley, California, United States of America; 3 Department of Epidemiology & Biostatistics, University of California San Francisco, San Francisco, California, United States of America; 4 Department of Pediatrics, University of California San Francisco, San Francisco, California, United States of America; 5 Philip R. Lee Institute for Health Policy Studies, University of California San Francisco, San Francisco, California, United States of America; Broad Institute of Harvard and MIT, United States Of America

## Abstract

While experimental and observational studies suggest that sugar intake is associated with the development of type 2 diabetes, independent of its role in obesity, it is unclear whether alterations in sugar intake can account for differences in diabetes prevalence among overall populations. Using econometric models of repeated cross-sectional data on diabetes and nutritional components of food from 175 countries, we found that every 150 kcal/person/day increase in sugar availability (about one can of soda/day) was associated with increased diabetes prevalence by 1.1% (p <0.001) after testing for potential selection biases and controlling for other food types (including fibers, meats, fruits, oils, cereals), total calories, overweight and obesity, period-effects, and several socioeconomic variables such as aging, urbanization and income. No other food types yielded significant individual associations with diabetes prevalence after controlling for obesity and other confounders. The impact of sugar on diabetes was independent of sedentary behavior and alcohol use, and the effect was modified but not confounded by obesity or overweight. Duration and degree of sugar exposure correlated significantly with diabetes prevalence in a dose-dependent manner, while declines in sugar exposure correlated with significant subsequent declines in diabetes rates independently of other socioeconomic, dietary and obesity prevalence changes. Differences in sugar availability statistically explain variations in diabetes prevalence rates at a population level that are not explained by physical activity, overweight or obesity.

## Introduction

Global diabetes prevalence has more than doubled over the last three decades, with prevalence rates far exceeding modeled projections, even after allowing for improved surveillance. Nearly 1 in 10 adults worldwide are now affected by diabetes [1]. This striking statistic has led to investigation into the population drivers of diabetes prevalence. Most of the worldwide rise is thought to be type 2 diabetes linked to the “metabolic syndrome” – the cluster of metabolic perturbations that includes dyslipidemia, hypertension, and insulin resistance. Obesity associated with economic development — particularly from lack of exercise and increased consumption of calories — is thought to be the strongest risk factor for metabolic syndrome and type 2 diabetes [2]–[5].

**Figure 1:**
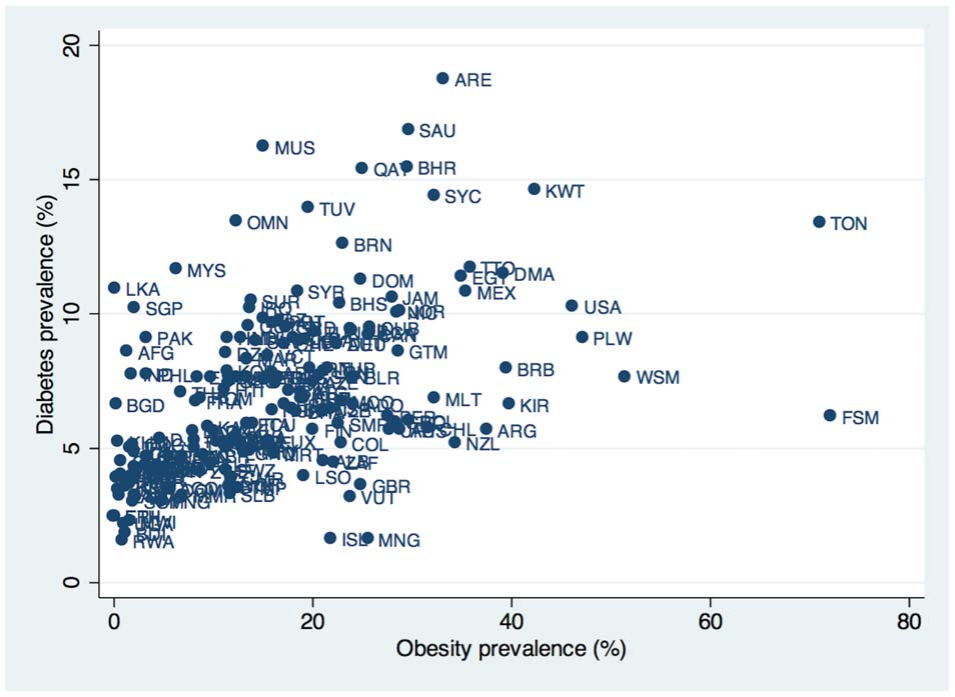
Relationship between obesity and diabetes prevalence rates worldwide. Obesity prevalence is defined as the percentage of the population aged 15 to 100 years old with body mass index greater than or equal to 30 kg/meters squared, from the World Health Organization Global Infobase 2012 edition. Diabetes prevalence is defined as the percentage of the population aged 20 to 79 years old with diabetes, from the International Diabetes Federation Diabetes Atlas 2011 edition. Three-letter codes are ISO standard codes for country names.

At a population level, however, obesity does not fully explain variations and trends in diabetes prevalence rates observed in many countries. As shown in **Figure 1**, several countries with high diabetes prevalence rates have low obesity rates, and vice versa. High diabetes yet low obesity prevalence are observed in countries with different ethnic compositions, such as the Philippines, Romania, France, Bangladesh and Georgia, although there are likely surveillance quality differences between nations [6], [7]. Trends in diabetes and obesity are also dyssynchronous within some nations; while Sri Lanka’s diabetes prevalence rate rose from 3% in the year 2000 to 11% in 2010, its obesity rate remained at 0.1% during that time period. Conversely, diabetes prevalence in New Zealand declined from 8% in 2000 to 5% in 2010 while obesity rates in the country rose from 23% to 34% during that decade. Similar trends of declining diabetes rates despite rising obesity rates were observed in Pakistan and Iceland. There are not obvious ethnic or socio-demographic commonalities between these countries to explain these observations. This population-level puzzle is accompanied by individual-level data. About 20% of obese individuals appear to have normal insulin regulation and normal metabolic indices (no indication of diabetes) and normal longevity [8], while up to 40% of normal weight people in some populations manifest aspects of the “metabolic syndrome” [9]–[12].

These findings direct attention to determining additional risk factors for development of diabetes. One controversial hypothesis is that excessive sugar intake may be a primary and independent driver of rising diabetes rates [13]. Sugars added to processed food, in particular the monosaccharide fructose, can contribute to obesity [14], but also appear to have properties that increase diabetes risk independently from obesity [15]. For example, liver fructose metabolism in the fed state generates lipogenic substrates in an unregulated fashion, which drives hepatic *de novo* lipogenesis and reduced fatty acid oxidation, forming excessive liver fat and inflammation that inactivates the insulin signaling pathway, leading to hepatic insulin resistance [16], [17]. Sugary foods have been significantly associated with the development of insulin resistance in laboratory-based studies [18], [19]. Reactive oxygen species are produced by the Maillard reaction [20], [21], damaging pancreatic beta cells, and leading to a subcellular stress response (the “unfolded protein response” in the endoplasmic reticulum) that drives insulin inadequacy [22], [23]. In concert, insulin resistance and reduced insulin secretion lead to overt diabetes.

Fructose is often consumed as high-fructose corn syrup (HFCS; 42% or 55% fructose) in the U.S., Canada, Japan, and some parts of Europe, while the rest of the world primarily consumes sucrose (50% fructose). Globally, countries have experienced a rise in sugar supply from an average of 218 kilocalories/person/day in 1960 to over 280 kilocalories/person/day today, with an acceleration in the rate of supply over the past decade. Assuming a 30% food wastage rate [24], these sugar calories exceed the recommended daily upper limit of 150 kilocalories per man and 100 kilocalories per woman suggested by the American Heart Association [25].

The issue of whether added sugars may be a population-level driver of the diabetes pandemic is of importance to global health policy. If obesity is a primary driver of diabetes, then measures to reduce calorie consumption and increase physical activity should be prioritized. However, if added sugar consumption is a primary driver, then public health policies to reduce sugar consumption warrant investigation as diabetes prevention proposals—especially for developing countries where diabetes rates are rising dramatically, irrespective of obesity.

In this study, we conducted a statistical assessment of panel data (repeated multi-variate data from multiple countries over a time period) to empirically evaluate whether changes in sugar availability, irrespective of changes in other foodstuffs, can in part account for the divergence in diabetes prevalence rates worldwide.

## Methods

We used United Nations Food and Agricultural Organization food supply data [26] to capture market availability of different food items (sugars, fibers, fruits, meats, cereals, oils, and total food) in kilocalories per person per day in each country for each year of the analysis. The dependent variables in the analysis were International Diabetes Federation estimates of diabetes prevalence among persons aged 20 to 79 years old from 2000 through 2010 [6]. We controlled for gross domestic product per capita (GDP expressed in purchasing power parity in 2005 US dollars for comparability among countries), percent of population living in urban areas, and percent of population above the age of 65 for each country in each year of the analysis from the World Bank World Development Indicators Database 2011 [27], and the prevalence overweight and obesity (percent of the population aged 15 to 100 years old with body mass index greater than or equal to 25 kg/m^2^ and 30 kg/m^2^, respectively) from the World Health Organization Global Infobase 2012 edition [7]. Data sources and summary statistics are further described in the Supporting Information (Text S1 and Table S1).

Data monitoring and quality was assessed through several approaches. First, a Hausman test [28] was performed to test whether factors that differ across countries such as the differing strength of diabetes surveillance systems would systematically affect our results, ensuring the available data were suitable to answer our research questions. This assesses for how reports of diabetes rates and food consumption may systematically differ between countries, so that such differences can be incorporated as controls in the statistical models. Selection bias may be an additional issue for assessing the effect of sugar on diabetes prevalence rates. Having greater sugar available in a country, for example, may be an artifact of overall economic development and increased general food importation, which could temporally overlap with rising diabetes prevalence irrespective of higher sugar intake (e.g., due to increased sedentary living or higher calorie intake leading to obesity). We controlled for this possibility using a lag of the change in log GDP per capita in our models. We also modeled the hazard of having high sugar availability rates in each country, and used this constructed hazard variable to explicitly control for potential unobserved selection bias (a “Heckman selection model”, see Text S1) [29]. We also used a set of period effects to control for secular trends in the diabetes and sugar data that may have occurred as a result of changes in countries’ diabetes detection capacity or sugar importation policies.

We conducted explicit model selection procedures using Generalized Estimating Equations (see results in Text S1) to ensure the model was an optimal choice for the given data [30]. The following regression model was specified, incorporating the leading factors believed to be related to diabetes prevalence, in addition to the sugar exposure variable:
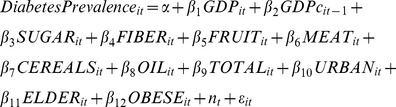
(1)


In Equation 1, *i* is country and *t* is year; GDP is logged per capita gross domestic product; GDPc is the lag of GDP change; SUGAR is the number of kilocalories per person per day of sugar availability (the sum of sugar, sugar crops, and sweeteners); FIBER is the number of kilocalories per person per day of fiber (constituting pulses, vegetables, nuts, roots and tubers); FRUIT, CEREALS, MEAT and OIL are the kilocalories per day per capita availability for each of these food categories; TOTAL is the total number of kilocalories per person per day of overall food availability; URBAN is the percentage of the country's population living in urban settings; ELDER is the percentage of the population that is age 65 or above; OBESE is the obesity prevalence rate; and *η* is the set of dummy variables which controls for period-effects, as described above; and *epsilon* is the error variable.

We subsequently added additional variables to test the associations of the percentage of total calories derived from sugar or other food components with diabetes prevalence, the duration of exposure to high calorie availability from sugar, and the effect of reduced sugar availability. We further tested the impact of introducing a measure of sedentary behavior, the estimated percentage of the population aged 15 years and older that is physically inactive from the International Physical Activity Questionnaire [31](defined as not meeting any of three criteria: (a) 5×30 minutes of moderate-intensity activity per week; (b) 3×20 minutes of vigorous-intensity activity per week; (c) an equivalent combination achieving 600 metabolic equivalent-minutes per week). Further control variables were the percent of persons above age 15 years who currently smoke tobacco, from the WHO Global Infobase [32], and the percent who engage heavy episodic alcohol drinking (at least 60 grams or more of pure alcohol on at least one occasion weekly), from the WHO Global Information System on Alcohol and Health [33].

We also performed Granger-causality tests, which use the temporal nature of the data to test whether high sugar availability preceded an increase in diabetes (“precedence”) or whether high diabetes prevalence preceded high sugar availability [34] (see Text S1). Data were analyzed in STATA v10.1. In all analyses, food availability data were age-adjusted, regressions were population weighted, and robust standard errors were computed to ensure stability of the results in the face of heteroskedasticity and intragroup correlations.

## Results

### Correlates of diabetes prevalence

**Table 1** presents the results of the cross-national model from 2000 to 2010. Each 150 kilocalorie/person/day increase in total calorie availability related to a 0.1% rise in diabetes prevalence (not significant), whereas a 150 kilocalories/person/day rise in sugar availability (one 12 oz. can of soft drink) was associated with a 1.1% rise in diabetes prevalence (95% CI: 0.48–1.7%; p<0.001) after all control variables were incorporated into the model. These controls included current income, changes in income, urbanization, aging, obesity, and the consumption of other foods as well as period effects (secular correlations that may have occurred simply due to surveillance changes or economic development). Diabetes prevalence rates rose 27% on average from 2000 to 2010, with just over one-fourth of the increase explained by a rise in sugar availability in this model. In countries like the Philippines, Romania, Sri Lanka, Georgia and Bangladesh, where high and rising diabetes rates were observed in the context of low obesity rates, sugar availability rose by over 20% during the study period. (It is possible that weight gain, rather than overt obesity, might account for some of the changes in diabetes, hence our models were repeated with overweight prevalence rather than obesity in Table S3, and with measures of physical inactivity rather than BMI in Table S4, but the results did not change).

**Table 1:** Effect of sugar availability on diabetes prevalence rates worldwide.

	(1)	(2)	(3)	(4)	(5)
	Diabetes prevalence (%)	Diabetes prevalence (%)	Diabetes prevalence (%)	Diabetes prevalence (%)	Diabetes prevalence (%)
Log GDP per capita	0.94^**^	0.86*	0.95*	1.00*	1.07*
	(0.33)	(0.37)	(0.37)	(0.40)	(0.48)
Change in log GDP	1.02	2.08	1.77	0.46	1.88
	(0.97)	(1.26)	(2.39)	(2.59)	(2.54)
Urbanization	0.048^**^	0.022	0.0048		0.016
	(0.015)	(0.013)	(0.011)		(0.011)
Aging	0.17*	0.11	0.039		0.049
	(0.067)	(0.081)	(0.075)		(0.085)
Total kilocalories		0.0010	0.00031	0.00079	0.00075
		(0.00056)	(0.00052)	(0.0012)	(0.0011)
Obesity			0.10^***^	0.094^***^	0.081^***^
			(0.024)	(0.022)	(0.021)
Sugar				0.0058^**^	0.0072^***^
				(0.0019)	(0.0020)
Fiber				0.00042	0.0011
				(0.0015)	(0.0014)
Fruit				0.00053	0.00011
				(0.0023)	(0.0024)
Meat				0.0032	0.0015
				(0.0023)	(0.0022)
Cereal				0.0014	0.0017
				(0.0013)	(0.0012)
Oils				0.00060	0.0018
				(0.0016)	(0.0018)
Countries	173	160	152	141	137
*R* ^2^	0.27	0.31	0.44	0.54	0.55

Food components are expressed in kilocalories/person/day, such that each row displays the impact on diabetes prevalence of a 1 kilocalorie/person/day increase in the availability of the given food category (e.g., a 1 kilocalorie/person/day rise in sugar relates to a 0.0072% rise in diabetes prevalence). Urbanization refers to the percentage of the population living in urban areas. Aging is the percentage of the population 65 years of age and older. Obesity is the percentage of the population with BMI at least 30 kg/m^2^.

Robust standard errors in parentheses.

* p < 0.05, ^**^ p < 0.01, ^***^ p < 0.001

Several of the main control variables in the model had important effects. The coefficient of log Gross Domestic Product (GDP) per capita was 1.07, which means that a 1% increase in GDP levels corresponded to a 1.07% rise in diabetes prevalence (p<0.05), consistent with the notion that economic development is a powerful correlate to diabetes prevalence [35], [36]. Similarly, variables capturing urbanization and aging populations were associated with diabetes prevalence; however these variables fell from significance as total food availability and obesity were incorporated into the model (**Table 1**), suggesting that calorie consumption and obesity are among the pathways by which these other factors may contribute to diabetes, consistent with cross-sectional studies [37].

A potential criticism of the basic finding is that, given the effect of obesity on the risk of diabetes and the high prevalence of both obesity and sugar availability in developed countries, our results are not due to sugar *per se* but rather confounded by rising obesity rates. In **Table 1**, we see that sugar availability remained a significant correlate to diabetes prevalence independent of obesity and total calorie consumption. When obesity was removed from the model, the effect size of sugar was not significantly amplified (beta  =  0.0081, p<0.001), suggesting that obesity does not appear to account for the major part of the impact of sugar on diabetes. We additionally tested whether sugar availability alone was a significant predictor of obesity rates independent of the other control variables (total consumption, urbanization, aging, income, other foods and period effects), and found the expected relationship between total calories and obesity, but not individually between sugar and obesity when total calories was accounted for—consistent with the hypothesis being tested (see Table S5).

None of the other food categories — including fiber-containing foods (pulses, nuts, vegetables, roots, tubers), fruits, meats, cereals, and oils — had a significant association with diabetes prevalence rates. We tested the hypothesis that low-carbohydrate fibers (nuts and vegetables) might be protective against diabetes by individually including them in the regression (as opposed to all fiber-containing foods) but they had no significant effect, and did not change the impact of sugar on diabetes prevalence. We initially separated fruit from other vegetables/fibers given the potential glucose burden of fruit; when repeating the analysis combining fruits with vegetables and other fibers, the results did not change.

### Tests of sugar exposure

As opposed to absolute sugar availability in kilocalories, the fraction of sugar in the available food market (the percent of total available calories composed of by sugar) may also be a critical factor in diabetes. As shown in **Table 2**, the fraction of total calories arising from sugar was the only significant food fraction correlated with diabetes, with a 1% rise in the fraction of total food calories as sugar corresponding to a 0.167% rise in diabetes prevalence.

**Table 2:** Fractional food composition and diabetes prevalence.

	(6)	(7)	(8)
	Diabetes prevalence (%)	Diabetes prevalence (%)	Diabetes prevalence (%)
Fraction of total calories from sugar	18.1^**^	15.7^**^	16.7^**^
	(5.53)	(5.16)	(5.41)
Fraction of total calories from fiber	3.97	1.00	1.70
	(2.98)	(3.24)	(3.37)
Fraction of total calories from fruit	–0.58	–1.98	–1.64
	(5.22)	(5.89)	(5.84)
Fraction of total calories from meat	3.97	9.31	7.82
	(7.01)	(5.89)	(5.89)
Fraction of total calories from cereal	0.96	2.27	2.73
	(2.97)	(2.99)	(3.07)
Fraction of total calories from veg oils	1.93	2.80	4.85
	(4.46)	(4.44)	(4.92)
Obesity	0.12^***^	0.092^***^	0.094^***^
	(0.029)	(0.021)	(0.021)
Log GDP per capita		1.03*	1.19*
		(0.44)	(0.47)
Change in log GDP		2.03	1.85
		(2.52)	(2.58)
Aging		0.036	0.036
		(0.086)	(0.087)
Urbanization			0.015
			(0.011)
Countries	147	137	137
*R* ^2^	0.49	0.54	0.55

Urbanization refers to the percentage of the population living in urban areas. Aging is the percentage of the population 65 years of age and older. Obesity is the percentage of the population with BMI at least 30 kg/m^2^.

Robust standard errors in parentheses.

* p < 0.05, ^**^ p < 0.01, ^***^ p < 0.001

We also tested whether the number of years a country was exposed to “high sugar availability”, which we defined as at least 300 kcal/person/day (twice the upper recommended daily limit for men, [25]) had a relationship with diabetes prevalence, by introducing a count variable for the number of years exposed to high sugar. Under the hypothesis being tested, longer exposure to sugar would correspond to greater effects on diabetes risk. We found that each extra year of exposure to high sugar availability was associated with an increase in diabetes prevalence of 0.053% (p<0.05) after all other control variables were included (**Table 3**).

**Table 3:** Years of sugar exposure and diabetes prevalence rates.

	(9)
	Diabetes prevalence (%)
Log GDP per capita	1.26^**^
	(0.46)
Change in log GDP	0.79
	(2.48)
Years of high sugar intake	0.053*
	(0.022)
Fiber	–0.0012
	(0.0012)
Fruit	–0.0022
	(0.0024)
Meat	0.0052
	(0.0031)
Cereal	0.00029
	(0.00099)
Oils	0.00015
	(0.0017)
Total kilocalories	0.0010
	(0.00099)
Urbanization	0.010
	(0.011)
Aging	0.082
	(0.084)
Obesity	0.099^***^
	(0.022)
Countries	137
*R* ^2^	0.52

Food components are expressed in kilocalories/person/day. Urbanization refers to the percentage of the population living in urban areas. Aging is the percentage of the population 65 years of age and older. Obesity is the percentage of the population with BMI at least 30x kg/m^2^.

Robust standard errors in parentheses

* p < 0.05, ^**^ p < 0.01, ^***^ p < 0.001

### Additional robustness checks

To further test whether influence runs from sugar availability to higher diabetes prevalence, and not vice versa (that is, to confirm that sugar availability did not increase as a result of whatever other factors associated with economic development or other unobserved variables may have raised diabetes prevalence), we tested the effects of lowering sugar availability. We found that in the periods after a country lowered its sugar availability (typically in the context of changes in trade agreements, discussed at length elsewhere, [38]), diabetes prevalence reduced by 0.074% (p<0.05), after correcting for changes in all other controls including the economic variables, socio-demographic variables, and changes in consumption of other food products as well as total calories and obesity prevalence (see Table S1).

We subsequently used Granger temporal causality tests (see Text S1) to test the robustness of this finding. We identified a significant relationship between high sugar availability and subsequently higher diabetes prevalence rates, not vice versa. Hence sugar availability did not violate criteria for temporal causality.

**Figure 2:**
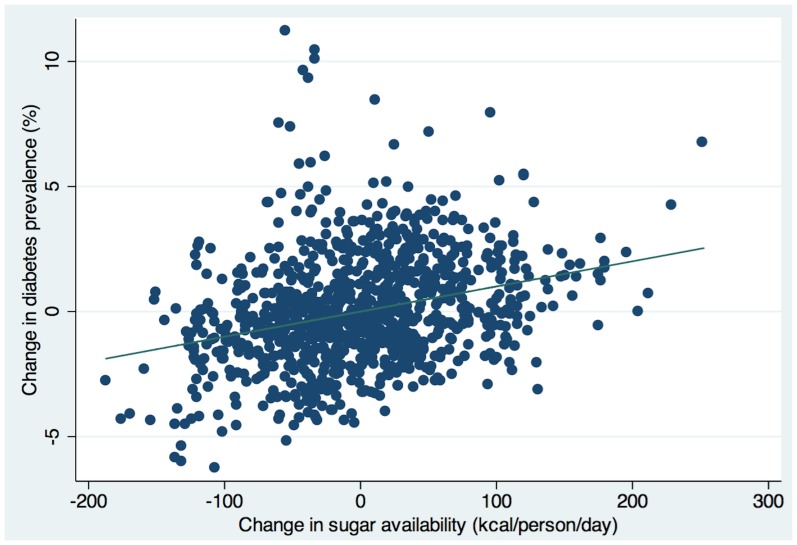
Adjusted association of sugar availability (kcal/person/day) with diabetes prevalence (% adults 20–79 years old). Regression line is adjusted for all control variables listed in Table 1, including time-trends (period-effects).

We conducted a series of additional robustness checks and regression diagnostics to test the sugar-diabetes relationship (see Tables S3, S4). **Figure 2** shows the plot of sugar availability and diabetes rates among all countries in the sample after control variables were introduced into the regression. First we removed potential outlying countries from this regression, liberally defined as countries having standardized residuals in the main model greater than the absolute value of 2. The results were strengthened: a 150 kcal/person/day rise in sugar availability corresponded to a 1.2% rise in diabetes prevalence (p<0.001) as opposed to a 1.1% rise when outliers were included. We also used other estimation approaches, including a time-series model that accounts for how earlier years in the regression may predict trends in later years and thereby throw off common regression models (an autoregressive time-series model using Stata’s xtregar module to explicitly estimate serial correlation), and the results remained significant: each 150 kcal/person/day rise in sugar availability related to a 0.4% rise in diabetes prevalence (p<0.001). We also re-ran these robustness checks with controls for country-specific factors (fixed effects) and without period effects, as well as using only direct diabetes survey data rather than some of the diabetes data that were imputed estimates by the International Diabetes Federation, and without the U.S. in the sample given a lower ratio of food consumption to supply in the U.S. than in other nations (higher food waste) [24]. In all cases, the sugar variable maintained a similar association with diabetes prevalence.

### Additional control variables

There are many additional epidemiological correlates to diabetes prevalence, and any econometric study is subject to limitations of data quality. We attempted to minimize any such potential confounding by introducing additional data measures and sources to test the robustness of our primary model. First, we reassessed our models using overweight (BMI ≥ 25 kg/m^2^) instead of obesity (BMI ≥ 30 kg/m^2^) in case obesity was a late-stage predictor of diabetes. We also incorporated physical inactivity, which has also been related to diabetes [39]. Lastly, a high prevalence of smoking and heavy alcohol use have been associated with diabetes [5]. Incorporation of these factors (see Table S4) did not affect the sugar variable and did not themselves reach statistical significance as independent correlates of diabetes when the other control variables were included in the model.

### Controlling for selection bias

These results may have been driven by another aspect of the changing environment for which we have not controlled. We addressed the issue of unobserved selection bias directly by constructing, and conditioning upon, a variable of the risk a country has of having high sugar availability (a first step bivariate probit model known as a “Heckman-type” selection model, see Text S1). Once we added controls for potential selection bias associated with high sugar availability, the association of sugar availability with diabetes prevalence magnified to 1.2% rise in diabetes prevalence for each 150 kcal/person/day increase in sugar availability (p<0.001). The coefficient on the variable for the risk of high sugar availability was non-significant, suggesting that selection bias was unlikely to impact our results.

## Discussion

The worldwide secular trend of increased diabetes prevalence likely has multiple etiologies, which may act through multiple mechanisms. Our results show that sugar availability is a significant statistical determinant of diabetes prevalence rates worldwide. By statistically studying variation in diabetes rates, food availability data and associated socioeconomic and demographic variables across countries and time, we identified that sugar availability appears to be uniquely correlated to diabetes prevalence independent of overweight and obesity prevalence rates, unlike other food types and total consumption, and independent of other changes in economic and social change such as urbanization, aging, changes to household income, sedentary lifestyles and tobacco or alcohol use. We found that obesity appeared to exacerbate, but not confound, the impact of sugar availability on diabetes prevalence, strengthening the argument for targeted public health approaches to excessive sugar consumption. We also noted that longer exposure to high sugar was associated with accentuated diabetes prevalence, while reduced sugar exposure was associated with decline in diabetes prevalence, and that the sugar-diabetes relationship appeared to meet criteria for temporal causality without being the result of selection biases or the effect of secular trends that may be artifacts of economic development or changes in surveillance.

Despite the robustness of our findings to a broad set of socioeconomic and epidemiologic variables, there are several important limitations to this analysis. First, as with all cross-country analyses, the potential exists for ecological fallacies. The observed associations are biologically plausible, given the numerous mechanisms by which sugar foments pathophysiologic processes leading to diabetes [19], [40]. They are also complemented by individual data, but unfortunately such individual analyses cannot identify what factors are most prominently affecting diabetes rates at the population level in the setting of multiple other concurrent economic and social changes. Hence, we add value to the discussion about diabetes prevention strategies by conducting an ecological statistical analysis that incorporates broad social change variables to assess the international significance of recent laboratory and clinical studies. An ecological analysis at a population level can also help decipher drivers of change from small associations found at the individual level. As an example, while not wearing bicycle helmets is found to be an important risk factor for traumatic brain injury in cohort studies, it is not an important driver of all traumatic brain injuries in general at a population level, since the latter is dominated by motor vehicle accidents. Similarly, in our analysis, many foods did not have significant correlations to diabetes prevalence at the population level, even though they are associated with diabetes in cohort or clinical trial studies. This is because at a population level the significance of these other foods may be not be driving population-level diabetes rates. Our population-level data do not allow us to assert mechanistic understandings of relationships between risk and outcome, but do afford us a sense that the effect size is large enough to affect the population rates of disease.

Second, we utilized an international food database that tracks caloric availability, as there are no direct measures of actual human consumption that can account for food wastage and provide precise measures of food consumption internationally. Exclusion of the United States from the data—an outlier-country in terms of food wastage—did not change our results. In other countries, supply and consumption are more closely aligned [41], and differential wastage among foodstuffs does not appear to occur [42]. Another potential limitation is that we cannot track specific foods with accuracy, hence further analyses should investigate and differentiate different types of sugars, or foods like dairy products, to which sugars are frequently added, as well as other nutritional components such as proteins and fats. For instance, a recent ecological analysis correlated high-fructose corn syrup with diabetes prevalence [43]. Our assessment was also ecological in nature and cannot identify specific longitudinal causation among individuals; however, unlike the prior assessment, the correlations detected here were subjected to several tests to assess relationships across time, the potential effects of other foodstuffs, the potential for selection biases, and a larger number of potential confounding factors.

Third, while considerable debate exists as to what forms of sugar may be most relevant to this relationship (for example, whether high-fructose corn syrup (HFCS) is different than sucrose [44]), our analysis cannot distinguish between any specific added sugars, such as HFCS or sucrose, or between any specific vehicle, such as soda or processed food. Our study merely suggests that the aggregate indicator of added sugar availability statistically predicts changes in diabetes prevalence over time.

Fourth, our ecological approach limits statistical power as one makes inferences about individuals based on aggregates; age, sex, and racial predictions are lost. Important work at the individual level suggests that certain populations, such as South Asian groups, may develop metabolic syndrome and diabetes at lower levels of obesity as assessed by BMI than other populations such as Caucasians. Environmental factors such as sugar consumption should be investigated as potential factors in this interaction. A BMI > 25 kg/m^2^ rather than 30 kg/m^2^ may a more appropriate indicator of obesity in Asians. Substituting overweight for obesity in the models did not change the effect size or significance of our findings with regard to sugar, and high sugars with low obesity rates were observed in countries outside of East and South Asia, suggesting that ethnic factors alone are unlikely to explain our observations. Other societal factors associated with diabetes were those classically associated with metabolic syndrome; including income, urbanization and aging. All three of these were associated with dietary and physical activity changes.

Finally, the International Diabetes Federation database contains diabetes prevalence data based on multiple surveys of varying quality; as many diabetics go undiagnosed, these are likely underestimates, and do not distinguish between Type 1 (approximately 10%) and Type 2 diabetes (90%), which would tend to produce regression towards the mean (underestimating the relationship between sugar and diabetes). Furthermore, we used the best available population-wide international data available to date for this assessment, but these data are known to be highly imperfect. It is thought that much of the FAO data on foods and nutrients in the food supply have limits to their reliability, and that IDF data and WHO data on obesity prevalence are difficult to validate independently. Hence, any of the findings we observe here are meant to be exploratory in nature, helping us to detect broad population patterns that deserve further testing through prospective longitudinal cohort studies in international settings, which are only now coming underway.

The observed relationship between dietary sugar exposure and diabetes in this statistical assessment was not mitigated by adjusting for confounders related to socioeconomics, aging, physical activity, or obesity. This suggests that sugar should be investigated for its role in diabetes pathogenesis apart from its contributions to obesity.

In summary, population-level variations in diabetes prevalence that are unexplained by other common variables appear to be statistically explained by sugar. This finding lends credence to the notion that further investigations into sugar availability and/or consumption are warranted to further elucidate the pathogenesis of diabetes at an individual level and the drivers of diabetes at a population level [13].

## Supporting Information

Table S1 **Summary statistics.**
(DOCX)

Table S2 **Lowered sugar availability and diabetes prevalence.**
(DOCX)

Table S3 **Replication of results using overweight instead of obesity.**
(DOCX)

Table S4 **Incorporating controls for physical inactivity, tobacco and alcohol.**
(DOCX)

Table S5 **Testing sugar as an explanatory variable for obesity.**
(DOCX)

Text S1 **Additional model information.**
(DOC)
