# Supplementary material for: The Relationship of Sugar to Population-Level Diabetes Prevalence: An Econometric Analysis of Repeated Cross-Sectional Data
Source: PLoS One. 2013 Feb 27;8(2):e57873. doi: 10.1371/journal.pone.0057873 (PMC3584048; doi:10.1371/journal.pone.0057873)
Supplement: Table S1 — Summary statistics. (DOCX) [file pone.0057873.s001.docx]

# Table S1. Summary statistics

| *Variable* | *Mean* | *SD* | *Min* | *Max* | *# of Countries* | *Source* |
| --- | --- | --- | --- | --- | --- | --- |
| Diabetes prevalence (% of adults 20 to 79 years old) | 6.39 | 2.99 | 1.39 | 19.49 | 204 | International Diabetes Federation |
| Log GDP per capita purchasing power parity (2005 $US) | 8.47 | 1.24 | 6.00 | 11.13 | 180 | World Bank |
| Change in Log GDP per capita, previous period | 0.02 | 0.02 | -0.05 | 0.11 | 179 | World Bank |
| Sugar availability (kcal/person/day) | 263.76 | 146.25 | 15.07 | 582.32 | 176 | Food and Agricultural Organization |
| Fiber availability (kcal/person/day) | 304.81 | 218.23 | 57.42 | 1231.16 | 176 | Food and Agricultural Organization |
| Cereal availability (kcal/person/day) | 1046.64 | 331.46 | 309.16 | 1834.88 | 176 | Food and Agricultural Organization |
| Meat availability (kcal/person/day) | 181.79 | 134.85 | 13.34 | 602.90 | 176 | Food and Agricultural Organization |
| Oil availability (kcal/person/day) | 204.10 | 113.68 | 23.16 | 571.25 | 176 | Food and Agricultural Organization |
| Fruit availability (kcal/person/day) | 100.63 | 71.04 | 1.54 | 448.86 | 176 | Food and Agricultural Organization |
| Total calorie availability (kcal/person/day) | 2527.14 | 475.60 | 1546.58 | 3710.27 | 176 | Food and Agricultural Organization |
| Obesity prevalence (% of adults 15-100 years old) | 14.28 | 11.57 | 0.05 | 69.86 | 184 | World Health Organization |
| Percent of population living in urban areas | 48.77 | 24.07 | 5.42 | 100.00 | 211 | World Bank |
| Percent of population 65 years of age or older | 5.94 | 3.78 | 1.50 | 15.96 | 191 | World Bank |
| Physical inactivity (% of population >15 years old) | 34.48 | 16.57 | 4.7 | 71.9 | 114 | Global Physical Activity Questionnaire |
| Daily tobacco smokers (% of population >15 years old) | 34.62 | 14.08 | 9.0 | 70.0 | 135 | World Health Organization |
| Heavy episodic alcohol drinking (% of population >15 years old) | 6.63 | 5.21 | 0 | 28.85 | 92 | World Health Organization |
