# Supplementary material for: The Relationship of Sugar to Population-Level Diabetes Prevalence: An Econometric Analysis of Repeated Cross-Sectional Data
Source: PLoS One. 2013 Feb 27;8(2):e57873. doi: 10.1371/journal.pone.0057873 (PMC3584048; doi:10.1371/journal.pone.0057873)
Supplement: Table S2 — Lowered sugar availability and diabetes prevalence. (DOCX) [file pone.0057873.s002.docx]

## Table S2. Lowered sugar availability and diabetes prevalence.

Food components are expressed in kilocalories/person/day. Urbanization refers to the percentage of the population living in urban areas. Aging is the percentage of the population 65 years of age and older. Obesity is the percentage of the population with BMI at least 30 kg/m^2^.

|  | (1) |
| --- | --- |
|  | Diabetes prevalence (%) |
| Change in log GDP per capita | 1.72^*^ (0.74) |
| Effect of lowering sugar availability | -0.074^*^ (0.036) |
| Change in fiber availability | 0.0000080 (0.00038) |
| Change in fruit availability | 0.0012 (0.0021) |
| Change in meat availability | 0.00017 (0.0011) |
| Change in cereal availability | -0.00042 (0.00051) |
| Change in oil availability | 0.00024 (0.00058) |
| Change in total consumption | 0.00027 (0.00048) |
| Change in urbanization | 0.47^***^ (0.12) |
| Change in elderly population | 0.75^*^ (0.32) |
| Change in obesity prevalence | 0.073 (0.079) |
| Observations | 141 |
| *R*^2^ | 0.10 |

Robust standard errors in parentheses

^*^ *p* < 0.05, ^**^ *p* < 0.01, ^***^ *p* < 0.001

## 
