# Supplementary material for: The Relationship of Sugar to Population-Level Diabetes Prevalence: An Econometric Analysis of Repeated Cross-Sectional Data
Source: PLoS One. 2013 Feb 27;8(2):e57873. doi: 10.1371/journal.pone.0057873 (PMC3584048; doi:10.1371/journal.pone.0057873)
Supplement: Table S3 — Replication of results using overweight instead of obesity. (DOCX) [file pone.0057873.s003.docx]

## Table S3. Replication of results using overweight instead of obesity.

Overweight is the percentage of the population with BMI at least 25 kg/m^2^.

|  | (2) | (3) | (4) | (5) | (6) |
| --- | --- | --- | --- | --- | --- |
|  | Diabetes prevalence (%) | Diabetes prevalence (%) | Diabetes prevalence (%) | Diabetes prevalence (%) | Diabetes prevalence (%) |
| Log GDP per capita | 0.94^**^ (0.33) | 0.86^*^ (0.37) | 0.91^*^ (0.40) | 0.92^*^ (0.43) | 1.05^*^ (0.50) |
| Change in log GDP | 1.02 (0.97) | 2.08 (1.26) | 2.00 (2.38) | 0.85 (2.57) | 2.29 (2.53) |
| Urbanization | 0.048^**^ (0.015) | 0.022 (0.013) | 0.0074 (0.011) |  | 0.018 (0.011) |
| Aging | 0.17^*^ (0.067) | 0.11 (0.081) | 0.064 (0.080) |  | 0.073 (0.093) |
| Total kilocalories |  | 0.0010 (0.00056) | 0.00043 (0.00051) | 0.0010 (0.0013) | 0.00084 (0.0012) |
| Overweight prevalence (%) |  |  | 0.055^***^ (0.013) | 0.046^***^ (0.013) | 0.038^**^ (0.012) |
| Sugar |  |  |  | 0.0066^***^ (0.0019) | 0.0078^***^ (0.0020) |
| Fiber |  |  |  | 0.00068 (0.0016) | 0.0013 (0.0014) |
| Fruit |  |  |  | 0.0014 (0.0025) | 0.00067 (0.0025) |
| Meat |  |  |  | 0.0025 (0.0024) | 0.00076 (0.0023) |
| Cereal |  |  |  | 0.0018 (0.0014) | 0.0019 (0.0012) |
| Oil |  |  |  | 0.00079 (0.0016) | 0.0021 (0.0018) |
| Observations | 173 | 160 | 152 | 141 | 137 |
| *R*^2^ | 0.27 | 0.31 | 0.42 | 0.51 | 0.53 |

Robust standard errors in parentheses

*^*^ p < 0.05, ^**^ p < 0.01, ^***^ p < 0.001*

## 
