# Supplementary material for: The Relationship of Sugar to Population-Level Diabetes Prevalence: An Econometric Analysis of Repeated Cross-Sectional Data
Source: PLoS One. 2013 Feb 27;8(2):e57873. doi: 10.1371/journal.pone.0057873 (PMC3584048; doi:10.1371/journal.pone.0057873)
Supplement: Table S4 — Incorporating controls for physical inactivity, tobacco and alcohol. (DOCX) [file pone.0057873.s004.docx]

## Table S4. Incorporating controls for physical inactivity, tobacco and alcohol.

|  | (7) | (8) | (9) |
| --- | --- | --- | --- |
|  | Diabetes prevalence (%) | Diabetes prevalence (%) | Diabetes prevalence (%) |
| Log GDP per capita | 1.98^*^ (0.79) | 1.98^*^ (0.91) | 1.80 (1.02) |
| Change in log GDP | 2.17 (6.85) | 4.59 (6.18) | 1.35 (8.13) |
| Sugar | 0.0080^*^ (0.0038) | 0.013^***^ (0.0029) | 0.014^**^ (0.0040) |
| Fiber | 0.00017 (0.0019) | 0.0030 (0.0019) | 0.00065 (0.0021) |
| Fruit | 0.0075 (0.0043) | 0.0071 (0.0044) | 0.0058 (0.0045) |
| Meat | 0.0044 (0.0028) | 0.0040 (0.0024) | 0.0061 (0.0031) |
| Cereal | 0.0022 (0.0014) | 0.0038 (0.0023) | 0.0030 (0.0019) |
| Oils | 0.0041 (0.0025) | 0.0074 (0.0053) | 0.0044 (0.0034) |
| Total | 0.00080 (0.0015) | 0.0030 (0.0023) | 0.0013 (0.0020) |
| Urbanization | 0.046^*^ (0.021) | 0.033 (0.020) | 0.031 (0.025) |
| Aging | 0.20 (0.12) | 0.23 (0.12) | 0.22 (0.14) |
| Physical inactivity (%) | 0.012 (0.019) |  |  |
| Tobacco smoking (%) |  | 0.019 (0.015) |  |
| Heavy alcohol use (%) |  |  | -0.020 (0.053) |
| Countries | 92 | 105 | 76 |
| *R*^2^ | 0.509 | 0.559 | 0.533 |

Robust standard errors in parentheses

*^*^ p < 0.05, ^**^ p < 0.01, ^***^ p < 0.001*

## 
