# Supplementary material for: The Relationship of Sugar to Population-Level Diabetes Prevalence: An Econometric Analysis of Repeated Cross-Sectional Data
Source: PLoS One. 2013 Feb 27;8(2):e57873. doi: 10.1371/journal.pone.0057873 (PMC3584048; doi:10.1371/journal.pone.0057873)
Supplement: Table S5 — Testing sugar as an explanatory variable for obesity. (DOCX) [file pone.0057873.s005.docx]

## Table S5. Testing sugar as an explanatory variable for obesity.

|  | (10) | (11) |
| --- | --- | --- |
|  | Overweight adults (%) | Obese adults (%) |
| Log GDP | 4.01^***^ (1.20) | 2.51^**^ (0.90) |
| Change in log GDP | 5.02 (3.03) | 1.03 (2.57) |
| Sugar | 0.0024 (0.0028) | 0.0022 (0.0025) |
| Fiber | -0.0044 (0.0026) | -0.0031 (0.0022) |
| Fruit | 0.011 (0.0069) | 0.011 (0.0073) |
| Meat | 0.0099 (0.0061) | 0.010 (0.0070) |
| Cereal | 0.0011 (0.0026) | 0.00074 (0.0023) |
| Oils | 0.0059 (0.0044) | 0.0041 (0.0037) |
| Total | 0.00063^*^ (0.0014) | 0.0014^*^ (0.0002) |
| Urbanization | 0.26^**^ (0.080) | 0.077 (0.054) |
| Elderly | 0.23 (0.26) | 0.34 (0.19) |
| Countries | 140 | 140 |
| *R*^2^ | 0.55 | 0.46 |

Robust standard errors in parentheses

*^*^ p < 0.05, ^**^ p < 0.01, ^***^ p < 0.001*
