# Supplementary material for: The Relationship of Sugar to Population-Level Diabetes Prevalence: An Econometric Analysis of Repeated Cross-Sectional Data
Source: PLoS One. 2013 Feb 27;8(2):e57873. doi: 10.1371/journal.pone.0057873 (PMC3584048; doi:10.1371/journal.pone.0057873)
Supplement: Text S1 — Additional model information. (DOC) [file pone.0057873.s006.doc]

# Text S1

# Model selection

To perform the statistical analyses, we noted that the dependent variable is characterized by nonnegative values only, has a positively skewed distribution (though the mean of 6.4 is not much larger than the median of 6.0), and is not likely to have truly independent values over time. This casts doubt on the appropriateness of ordinary least squares regressions. We therefore tested our model against the broader class of Generalized Estimating Equations (GEE), which do not require the assumptions of ordinary least squares regression and can explicitly incorporate the lagged-dependent nature of the data (auto-regressivity). GEE models are an extension of generalized linear models that use a link function g() to relate the expected value of the dependent variable y to a linear function of the explanatory variables and specifies a variance distribution F() for y, such that g{E(y)}=x’β where y ~ F(y). To assess the fit of different link functions g and distributions F using standard approaches, we used comparisons of the Akaike Information Criterion (a test of which model fits the data with minimal complexity); the modified Park/GLM family tests; Pregibon’s link test (which assesses the linearity of the mean function x' β); and a modified Hosmer-Lemeshow test for systematic misspecification (which divides the data into deciles based on x'β and conducts an F-test to determine whether the mean residuals across groups differ from zero) (1). We found no significant advantages comparing the model with identity link and Gaussian distribution (the default model presented in the main text and specified below) to the inverse Gaussian, negative binomial, Poisson or gamma variance distributions F with a log or identity g link and either compound symmetry for the correlation structure, autoregressivity or an unstructured correlation matrix. We nevertheless re-ran the regressions using the log link and Gaussian, Poisson and Gamma variance functions to assess the robustness of the main model findings; none of the results significantly changed.

# Granger causality test

We found that diabetes prevalence was a function of lagged sugar availability, Diabetes*_t_* = βSugar*_t-1_,* and not the other way around (i.e., we found no association in the following regression: Sugar*_t_* = βDiabetes*_t-1_*).

In standard usage, a variable X is said to “Granger cause” another variable Y if Y can be better predicted from the past of X and Y together than the past of Y independently. Note that “Granger causality” is not proof of true causality, but actually refers to the predictive ability of one variable on another. “Granger causality” tests “precedence”, i.e., whether Y precedes X, X precedes Y or they are contemporaneous (3).

The Granger test is specified as:

Y_t_ = α_1_Y_t-1_ + β_1_X_t-1_ + μ_t_

As shown in the table below, we found that higher sugar availability Granger-causes higher diabetes prevalence but that diabetes prevalence fails to Granger-cause sugar availability. The same pattern was not observed with obesity and diabetes, suggesting that while sugar passes the Granger test for temporal causality, obesity fails (possibly, as discussed in prior literature, due to the fact that obesity is a correlate or marker of diabetes rather than a long-term early driver) . The choice of lag length is to some degree arbitrary (2), but the findings above remained robust as we varied the lag length across the range of available observations.

| Granger Causality Tests | | | | |
| --- | --- | --- | --- | --- |
|  | Dependent Variable | | | |
| Covariate | Sugar*_t_* | Diabetes prevalence*_t_* | Obesity prevalence*_t_* | Diabetes prevalence*_t_* |
| Diabetes prevalence*_t-1_* | -0.19 (0.31) | 0.93^***^ (0.0062) | 0.011 (0.030) | 0.98^***^ (0.0049) |
| Sugar*_t-1_* | 0.98^***^ (0.0043) | 0.00066^***^ (0.000085) |  |  |
| Obesity prevalence*_t-1_* |  |  | 1.02^***^ (0.00082) | 0.00042 (0.0013) |

*Significance at * p<0.05, ** p<0.01, *** p<0.001*

# Heckman model

Selection bias involves non-random assignment to a treatment and control group; that is, there is some unobserved factor relating to having high sugar availability that is also related to high diabetes prevalence in a way we have not observed and as a result for which we have not corrected.

We can address the issue of unobserved selection bias directly by constructing, and controlling for, a variable of the “hazard” of having high sugar availability (a “Heckman-type” selection model or “control function” approach) (4).

In medicine, randomized controlled trials include a random assignment pattern that can identify the unbiased average effect of an intervention. In observational studies, a Heckman selection model or “control function” approach can be used to model the selection into a “high-sugar-availability group” based on a set of unobservable and observable factors, and then correct for this selection in the outcome equation.

To do this, we define a system of equations:

(1) Diabetes_it_ = α_1_ + β_1_SUGAR_it_ + β_2_X_it_ + β__λ_it_ + μ_i_ + ε_it_ (the “outcome equation”)

and

(2) SUGAR_it_ = α_2_ + β_4_X_it_ + β__W_it_ + μ_it_ (the “selection equation”)

Here, X is the set of controls used in the main equation 1, *λ *is a selection coefficient (defined below), and W is a set of variables that affect sugar availability but are not included as predictors of diabetes prevalence (for example, the percentage of food that is imported is a significant determinant of sugar availability but is not used as a predictor of diabetes prevalence). The selection equation is a bivariate probit model in which high sugar availability was defined as at least 300 kcal/person/day (twice the recommended daily intake limit for men), though the results below remained robust when the definition of high availability was varied between 200 and 600 kcal/person/day.

A selection coefficient can be calculated as λ = φ(Z)/(1 − Φ(Z)), where predictions of high sugar availability from the selection equation give the standard normal cumulative distribution function Φ(Z), from which we can calculate the normal distribution φ(Z) of these predictions using the Gaussian function e-(ρ^2/2)/√(2Π). Since hazard has the general definition H(x) = φ(Z)/(1 − Φ(Z)), we are simply controlling for the “hazard of high sugar availability” in our models; if the selection coefficient has a significant relationship to diabetes prevalence, this would suggest that selection bias may be a problem for our model. As shown in the table below, the selection coefficient did not have a significant relationship to diabetes prevalence.

Key assumptions for applying the Heckman approach are: a) joint bivariate normality (that is, ε ~ N(0, σ) and μ ~ N(0, 1)) (5); and b) a need for some variables in W are used to identify Z that are not in the set of variables X (6). We identified food imports as a percentage of overall imports as such an instrumental variable. The criteria for an instrumental variable *w* to be valid are that first, it has to be correlated with the variable of interest (Cov(*w*, Sugar) ≠0), which can be tested, and second it has to be uncorrelated with heterogeneity in the outcome equation (Cov(*w*, ε) = 0), which cannot be directly tested. We directly tested that the first condition holds (Cov (food imports, sugar)=0.26, p<0.001). A diagnostic test for the second requirement was performed using a test for overidentification, which involves regressing the residuals from the outcome equation on the instrument set. Our tests rejected the null hypothesis that the instruments are correlated with the error term of diabetes equation, which strengthens the case that our instrument is valid.

Of note, while the Heckman-selection strategy is a standard approach to evaluating models for selection bias, it features important limitations, including sensitivity to the model specification. For these reasons, we regard the selection model as an additional robustness check on our basic finding, not as definitive “proof” that selection bias is not still possible in our experiment, as we have additionally specified in the main manuscript.

| Association of Sugar Availability (kcal/person/day) with Log Diabetes Prevalence, Adjusted for Selection Bias | |
| --- | --- |
| Effect of Sugar Availability | 0.008 (0.0009)*** |
| Effect of Sugar Hazard Rate *λ* ^†^ | -1.08 (0.66) |

* *p<0.05, ** p<0.01, *** p<0.001*

*Note:* Robust standard errors in parentheses clustered by country. Models include controls used in the basic specification in equation 1.

† *λ* is the hazard rate of sugar availability, also referred to as the Inverse Mills Ratio.

# References

1. Hardin JW, Hilbe JM (2003) Generalized estimating equations. Boca Raton: CRC Press.

2. Maddala G (1983) Limited-Dependent and Qualitative Variables in Econometrics. Cambridge: Cambridge University Press.

3. Bremer AA, Mietus-Snyder ML, Lustig RH (2012) Toward a unifying hypothesis of metabolic syndrome. Pediatrics 129: 557-570.

# 
